# To Make Leeches Bite Promptly

**Published:** 1877-07

**Authors:** 


TO MAKE LEECHES BITE PROMPTLY.—Place the leeches in a glass half full of cold water. Cleanse the part to which they are to be applied carefully with warm water, and then apply the glass containing the leeches to the part. They attach themselves with surprising rapidity. The patients often speak of the bites appearing to be simultaneous. When the animals have all become attached, allow the water to escape into a sponge or cloth, so as not to wet the patient.—*Gazz. Med. Ital. Lomb.*

